# Strengthening Collaborations in Latin America for Advancing Oral cancer research and public health policies

**DOI:** 10.4317/jced.62884

**Published:** 2025-07-01

**Authors:** Juliana L. Schussel, Mariana Villarroel-Dorrego, Sven Eric Niklander

**Affiliations:** 1 Post Graduation Program in Dentistry, Department of Stomatology, Universidade Federal do Paraná; 2 Institute of Dental Research, School of Dentistry, Central University of Venezuela, Caracas, Venezuela; 3 Unit of Oral Pathology and Medicine, Dentistry faculty, Universidad Andres Bello, Chile

## Abstract

**Background:**

Oral cancer (OC) represents a significant public health challenge in Latin America, with similar challenges presented in most countries of the region. Regional partnerships are needed, as they can enhance the development of educational campaigns, prevention strategies addressing shared risk factors, and institutional strengthening. Additionally, expanding collaborations can attract international funding and integrate LAC researchers into global initiatives.

**Material and Methods:**

This review examines the state of OC research in the region, highlighting collaborative efforts, gaps, and opportunities for advancement.

**Results:**

We identified 17 studies that have studied different aspects of oral cancer in Latin American countries (LAC). Studies varied in design, with ecological studies and case series being the most common. Most studies focused purely on OC, while research on oral potentially malignant disorders (OPMD) remains limited. Collaborative studies have increased over the past decade, with a growing focus on regional risk factors such as tobacco and alcohol use. Despite these advances, challenges persist, including limited epidemiological data, underreporting of cases, and inadequate public health infrastructure.

**Conclusions:**

Collaborative networks have the potential to reduce these gaps by pooling resources, expertise, and data. Improved public health policies supported by robust local data are crucial for reducing the OC burden in LAC. Strengthening regional and international collaborations will be fundamental for the advancement of OC research, improving early detection, and implementing effective prevention and treatment strategies tailored to the region’s unique challenges.

** Key words:**Oral cancer, mouth neoplasms, Latin America, oral potentially malignant disorders.

## Introduction

In recent years, there has been a significant increase in collaborations between Latin American countries (LAC) in research, especially on oral cancer (1). This movement has been crucial for scientific advancement and the development of regional strategies to combat the disease. The socio-economic and cultural similarities among Latin American countries provide a common foundation that facilitates the creation of research protocols and the implementation of public policies specific to the needs of the region. However, despite this progress, expanding these collaborations even further is essential to address the remaining gaps in research and improve public health outcomes.

The aim of this paper was to perform a review of the situation of oral cancer research in LAC, by identifying studies that have been done in this area.

## Material and Methods

To identify studies related to oral cancer (OC) and/or oral potentially malignant disorders (OPMD) focusing on the Latin-american region, we used the terms ((latin america) AND (oral cancer)) OR (oral precancer) OR (oral potentially malignant disorder). A manual search was also performed. Inclusion criteria were review articles, position papers, observational studies, systematic reviews, case series related to any type of oral cavity cancer in Latin America. Studies of head and neck cancer were included only when specifying that cases of OC were part of the cohort. There was no time limit. Exclusion criteria were case reports, and Latin-American studies about cancers of anatomical regions different than the oral cavity.

## Results

The search identified a total of 956 studies, 10 of which were duplicates. The records were filtered by title and abstract, and 916 articles were eliminated as were not related to the topic. Finally, 30 articles were fully reviewed, and 17 were included ( Table 1).

Of the 17 records, 14 had representation of more than 2 different countries (range 1 to 23) with an average of 4,9 countries per record. Brazil was the country with the highest number of participations, being involved in 16 of the 17 articles, followed by Colombia (n= 7), Mexico (n= 7), Argentina (n =6), Perú (n= 6) and Chile (n= 5). All other countries were involved in 3 or less articles. According to the year of publication, 2 studies were published between 2001-2008, 6 between 2010-2019, and 8 between 2020-2024. Twelve studies declared to study oral cancer but only one specified to be OSCC, and in 2 studies oral cancer was included in as a subset of a HNSCC cohort. There were only 4 studies that focused on OPMD; 1 was a systematic review, 1 was survey, 1 assessed the frequency of OSCC associated with OPMD, and 1 a case series of HPV-OED. Apart from that last paper, none of the others specified which OPMD was studied. There was 1 study about primary oral melanoma and 1 case series of malignant odontogenic tumors. The most common study design was Ecological study (n= 3) and case series (n= 3), followed by review articles (n= 2), case-control studies (n= 2) and Cross-sectional studies (n= 2). We identified no clinical trials ( Table 1).

## Discussion

The incidence of cancer in LAC is high and projected to increase, being one of the most frequent causes of death for most countries (2,3). Although there is a significant variability in cancer burden in LAC, similar challenges are present in most countries such as socioeconomic conditions and access to therapies (3) 

LAC economies are growing rapidly, leading aging of the population and an increase in lifestyles unhealthy patterns. Implementation of control policies of risk factors for oral cancer, such as tobacco and alcohol, have proven to have an effect on oral cancer mortality, showing the need for more studies that can improve preventive measures.

In most of LAC, the main risk factors causing diseases are tobacco, alcohol consumption and high rate of body mass. Tobacco is one of the main risk factors for death and disability, contributing to poverty and exerting an enormous economic burden on health systems. Smoking is responsible for 351,000 deaths per year in LAC, causing $22.8 billion in direct medical costs, $16.2 billion in lost productivity, and $10.8 billion in caregiver costs (4). There are currently 22 countries of the region that are implementing measures to advise about the dangers of tobacco use through graphic health warnings on packaging of tobacco products. In Brazil, starting in 1996, there was a progressive restriction on cigarette advertising and the adoption of significant control measures. In 2019, Uruguay enacted policies that now require plain packaging of tobacco products. Antigua and Barbuda (2018), Venezuela (2019) and Mexico (2021) have prohibited advertising, promotion and sponsorship of tobacco products (5). Despite all this, only 10 countries have surveillance systems that provide recent, periodic and representative data on tobacco consumption in adults and youth. Additionally, there is no data on secondhand smoke exposure, making it difficult to accurately assess the impact of smoke-free policies. There remains a gap in expanding access to tobacco cessation services, such as helplines, medications and behavioral therapies (4).

LAC continues to face significant challenges in oral cancer research, especially regarding epidemiological data. The region lacks comprehensive national registries and large-scale studies are needed, making it difficult to assess the true incidence and prevalence of the disease (6). We identified only 3 large-scale ecological studies (2,7,8). The underreporting of oral cancer cases in LAC limits the ability to develop precise public health and may prevent the achievement of the primary prevention goals (9). An important effort to improve prevention and information in the region is the Latin American and Caribbean Code against Cancer Framework, a collaboration of the Pan American Health Organization (PAHO) and the International Agency for Research on Cancer (IARC), that gather information from LAC and aims to reduce the burden of cancer in the region (https://cancer-code-lac.iarc.who.int/es/).

Collaborative efforts across countries can help fill these gaps by pooling data, resources, and expertise, which will lead to more effective, localized health interventions. Additionally, many LAC share common risk factors for oral cancer, such as high rates of tobacco use, alcohol consumption, poor access to preventive dental care, and socioeconomic aspects. By improving collaborative research efforts, countries in the region can design more targeted prevention strategies aimed at addressing these shared risk factors. This will not only lead to more effective interventions but will also help public health policies in the region.

Brazil is by far the country that has contributed the most in oral cancer research in Latin America. Brazilian researchers were involved in 16 of 17 identified studies. In addition to being the largest country in the region with the highest population, several factors also contribute to the prominence in research, such as the high incidence of the disease, a well-established network of scientific research centers that has been enhanced over the years with public funding, and the Unified Health System (SUS), one of the world’s largest public healthcare systems, which provides robust epidemiological data. This strong presence in research highlights Brazil’s important role in fostering integration and collaboration among other Latin American countries.

The lack of public health literacy, particularly regarding the early symptoms and risk factors of oral cancer, is an important challenge for its prevention and diagnosis. Strengthening collaborative networks can help the development of educational campaigns designed to enhance health literacy and awareness among both the general population and healthcare professionals, while addressing the linguistic and cultural diversity within the region. (10). Many countries in the region face a shortage of healthcare professionals trained in the diagnosis and treatment of oral cancer (6). Through collaborative networks, joint training programs, workshops, and academic partnerships can support the training and capacity-building of healthcare professionals, equipping them to be better prepared for addressing the challenges posed by the disease. This will ensure a more prepared workforce to address the rising cases of oral cancer and improve the quality and reliability of data and studies emerging from the region (10).

Discussions on oral potentially malignant factors (OPMD) and its management also seems to be barrier for early diagnosis, and regional risk factors must also be taken into account for screening (6,11). We identified only 4 collaborative studies on OPMD, which is probably a reflection of the lack of registries regarding this topic in LAC. It seems that the new generations of oral pathologists and oral medicine specialists of the region are conscient of this issue, as all 4 articles available were published between 2023- and 2024. Nevertheless, there is a lack of data regarding the types, frequency, regional risk factors, and malignant transformation rate of OPMD in the region.

Access to funding and resources is another critical factor. Besides socioeconomic factors, economical investments also influence the mortality rate of cancer in LAC (12).Thus, international collaborations can help attract funding from global health organizations, foundations, and governments to support research design for the specific needs of the region. Presenting a united front and demonstrating the potential for regional impact increases the chances of financial support. Moreover, expanding intra-regional collaborations would also facilitate Latin America’s integration into global research networks. By participating in international initiatives and conferences, such as the World Workshop on Oral Medicine, Latin American researchers can share knowledge with experts from around the world and ensure that the region’s unique challenges are included in global discussions about cancer prevention and treatment. Importantly, the results from collaborative regional research can serve as a strong foundation for advocating public health policies. Local data and evidence generated through collaborative studies will enable policymakers to implement more effective, evidence-based strategies for the prevention, early diagnosis, and treatment of oral cancer. It also ensures that best practices are shared and applied across borders, strengthening public health responses throughout the region.

Finally, these collaborative efforts will contribute to reducing healthcare disparities in the region. By focusing on underserved populations, such as those in rural areas or with limited economic resources, regional collaborations can promote more equitable access to care, ensuring that advancements in research and public health interventions benefit all segments of the population. Therefore, strengthening collaborations between Latin American countries is essential for advancing oral cancer research and improving public health outcomes in the region. Collaborations in the region have significantly increased during the last decade, but more efforts have to be done. By expanding these partnerships, countries can address gaps in data, improve capacity, secure funding, and ultimately reduce the burden of oral cancer, leading to improved quality of life and reduced health disparities throughout Latin America.

## Figures and Tables

**Table 1:** Identified records that have studied different aspects of OC and/or OPMD in Latin America.

Year	Authors	Nº countries	Countries	Lesion	Nº	Type of study	Aim	Conclusion
2024	MartÃ­nez-RamÃ­rez et al. (6)	21	Brazil, El Salvador, Colombia, MÃ©xico, Uruguay, Argentina, PerÃº, Jamaica, Indonesia, Chile, Venezuela, Paraguay, Guatemala, Cuba, Belize, Honduras, Santo Domingo, Nicaragua, USA, France, UK	OC , OPMD	n= 23 participants	Cross-sectional-survey	To explore perceived barriers to early diagnosis and management of oral cancer and potential pathways for improvement in Latin America and the Caribbean	Identification of barriers and recommendatios for improvement
2021	Freire et al. (12)	1	Brazil	OC	N/A	Cross-sectional retrospective	To analyze the influence of socioeconomic indicators and economical investments on oral cancer mortality rates in Latin American countries	Socioeconomic factors and economical investments influence the mortality rate of oral cancer in Latin American countries.
2024	Matos et al. (13)	11	Brazil, PerÃº, Uruguay, Argentina, Colombia, Canada, Chile, MÃ©xico, USA, Scotland, France	HNSCC	n= 41 participants	Consensus-delphi survey	To establish Latin American guidelines for the care of patients with HNSCC considering	Establishmetn of 48 recommendations on care of patients with HNSCC considering the availability of resources and focusing on oncologic benefit in the reality of Latin America.
2022	Serna et al. (2)	2	Colombia, Brazil	OC	N/A	Ecological	To describe the trend in incidence, mortality and Disability Adjusted Life Years (DALYs) of oral cancer in Latin America	Oral cancer shows increasing trends in: the incidence in both sexes in 10 countries, in mortality and DALYs in 6 countries.
2019	Herrera-Serna et al. (8)	4	Colombia, MÃ©xico, Brazil, Chile	OC	n= 23.951 OC	Ecological	To evaluate the relationship between the Human Development Index (HDI) and its components with oral cancer (OC) in Latin America	Oral cancer incidence and mortality rates vary both among and within Latin American countries according to sex, with a greater burden on men.
2001	WÃ»nsch-Gilho et al. (14)	1	Brazil	OC	N/A	Review	To examine incicence and mortality rates according to risk factores of mouth cancer in Latin America and the Caribbean	Mouth and oropharyngeal cancer in Latin America and the Caribbean is a huge public health problem.
2024	Gilligan et al.(11)	8	Argentina, Brazil, PerÃº, Costa Rica, MÃ©xico, Chile, Venezuela, Uruguay	OSCC, OPMD	n= 2705 OSCC	Retrospective observational	To determine the frequency of oral squamous cell carcinoma (OSCC) associated or not with oral potentially malignant disorders (OPMD).	In Latin America, de novo OSCC seems to be more frequent with regional variations of some clinical and histopathological features
2024	Pedrosco et al. (10)	2	Brazil, UK	OC, OPMD	N/A	Systematic review	To assess the outcomes of oral cancer screening in the Latin American region	Screening studies conducted in Latin American countries had serious limitations both in methodology (lack of examiner training) and in reporting data (lack of description of clinical categories of screen positives.
2022	Sichero et al. (15)	6	Brazil, Italy, PerÃº, Argentina, Colombia, France	HNSCC	n= 1375 HNSCC	Retrospective	To evaluate HPV DNA in FFPE HNSCC from Argentina, Brazil, Colombia and Peru	HPV-16 might have a role in a subset of HNSCC. There is heterogenity between samples from different countries.
2012	de-Andrade et al. (16)	4	Brazil, Guatemala, PerÃº, MÃ©xico	POM	n= 22 POM	Retrospective	To analyze the histopathological and immunohistochemical characteristics of primary oral melanomas	S-100 and HMB-45 are more frequently expressed than Melan-A in primary oral melanomas
2019	Herrera-Serna et al. (7)	3	MÃ©xico, Colombia, Brazil	OC	n= 103.134 OC	Ecological	To describe the trends of mortality from oral cancer between 2000 and 2017, by sex, in 20 countries in Latin America.	The effect of the implementation of control policies is evidenced by a greater relationship with oral cancer mortality in the countries with the least progress in their execution.
2014	MartÃ­nez et al. (17)	4	Brazil, MÃ©xico, Guatemala, PerÃº	MOT	n= 25 MOT	Retrospective	To show the epidemiological features of 25 malignant odontogenic tumors in Latin America	This series helps to better clarify the relative frequency, predominant subtypes, and clinical characteristics of MOTs in Latin America.
2024	Santos-Silva et al. (18)	1	Brazil	OC	N/A	Letter to editor	N/A	There is an underreporting of Oral cancer in Latin America and the Caribbean.
2008	Parkin et al. (19)	4	UK, Spain, France, Colombia	OC and genitalia	N/A	Review	To present the burden of human papillomavirus (HPV)-related cancers in terms of incidence and mortality, for the countries of the Latin America and Caribbean region	Approximately 65% of cervical cancer cases and 50% of the high risk lesions are associated with HPV-16 and 18. Incidence rates of other HPV-related cancers are significantly lower
2011	Szymanska et al. (20)	4	Brazil, Argentina, Cuba, Canada	OC, OPC, LC, EC	n= 2252 (OC, OPC, LC, EC) controls= 1707	Case-control	To assess the role of tobacco and alcohol consumption as well as their interactions in the development of squamous-cell carcinomas of the four sites of the upper aerodigestive tract in high-risk populations in Latin America	The majority of cancer cases of the upper aerodigestive tract are due to a combined effect of alcohol and tobacco use and could be prevented by quitting the use of either of these two agents
2010	Szymanska et al. (21)	5	Argentina, Canada, Brazil, USA, France	OC, OPC, LC, EC	n= 1168 (OC, OPC, LC, EC), n= 1026 controls	Case-control	To assess the role of mate consumption in the development of squamous-cell carcinomas of the four organs of the upper aerodigestive tract in high-risk populations of South Americ	The consumption of mat increases the risk of esophageal cancer but not necessarily other cancers of the upper aerodigestive tract
2023	Roza et al. (22)	2	Brazil, Chile	HPV-OED	n= 5 HPV-OED	Case series	To report 5 cases of HPV-associate oral dysplasia from Latin America	Further larger and well-documented series are needed to expand the knowledge of HPV-OED, including its biological behavior in HIV-positive patients.

N/S= not stated, N/A = not applicable, OC= Oral cancer, OPMD= Oral potentially malignant disorder, FFPE= Formalin fixed paraffin embedded, HNSCC= Head and neck squamous cell carcinoma, POM= primary oral melanoma, MOT= malignant odontogenic tumors, OL= oral leukoplakia, OE= oral erythroplakia, OPC= oropharyngeal cancer, LC= laryngeal cancer, EC= esophageal cancer, HPV-OED= HPV associated oral epithelial dysplasia.

## Data Availability

The datasets used and/or analyzed during the current study are available from the corresponding author.
